# Transradial versus transfemoral approach for TACE: a retrospective study

**DOI:** 10.1186/s12876-023-02646-1

**Published:** 2023-01-11

**Authors:** Ke You, Tao Guo, Da Sun, Hao Song, Zuojin Liu

**Affiliations:** 1 Department of Hepatobiliary Surgery, The Second Affiliated Hospital of Chongqing Medical University, Linjiang Road 74, Chongqing, 400010 China; 2 Liuzhou Key Laboratory of Infection Disease and ImmunologyGuangxi Health Commission Key Laboratory of Clinical Biotechnology, Liuzhou People’s Hospital affiliated to Guangxi Medical University, Liuzhou, 545006 China; 3 Department of Pathophysiology, School of Basic Medical Sciences, Weifang Medical University, Weifang, 261053 China; 4 Department of Organ Transplantation, Third Affiliated Hospital of Naval Military Medical University, North Moyu Road 700, Shanghai, 201805 China

**Keywords:** Transradial access, Transfemoral access, TACE

## Abstract

**Objective:**

Transcatheter arterial chemoembolization (TACE) has been widely applied in the treatment of hepatocellular carcinoma (HCC). Our study aimed to investigate the feasibility and efficacy of transradial access as an alternative to transfemoral access for TACE.

**Methods:**

Patients undergoing TACE were divided into the radial artery (RA) route group or the femoral artery (FA) route group according to the operation approach, namely, transradial or transfemoral access. We retrospectively analysed the clinical characteristics, technical outcomes, clinical efficacy and incidence of adverse events to compare the two technologies for intervention for HCC.

**Results:**

Transradial access was found to achieve superior technical outcomes and clinical efficacy, as the patients in the RA group had a lower rate of hepatic arterial spasm, a higher partial response rate and a lower progression rate than the patients in the FA group according to the mRECIST evaluations. In contrast, the liver function indices and VAS (visual analogue scale) pain scores were consistent across the two groups. Moreover, patients in the RA group had a shorter length of stay than those in the FA group, despite similar hospitalization expenses. The total adverse events were significantly reduced by transradial access for TACE (72.5% vs. 84.1%, *P* = 0.027).

**Conclusion:**

Our study suggested that transradial access is an effective and feasible alternative to transfemoral access for TACE. Large-scale prospective randomized controlled studies are expected.

## Introduction

Primary hepatocellular carcinoma (HCC) is often diagnosed at advanced stages as a result of insidious onset; thus, it is imperative to develop nonsurgical therapies for unresectable tumours. Transcatheter arterial chemoembolization (TACE) is an extensive and effective approach for HCC and is recommended in most clinical guidelines as a superior treatment for unresectable hepatocellular carcinoma [1–4].

Traditional TACE is based on the percutaneous catheterization technique developed in 1953 [5] and is performed through femoral arterial access, which has been extensively applied in many catheterization interventions [6, 7]. Progress has been made in the development of technologies and materials, and angiography and intervention via transradial access to the upper limbs have been recently developed for coronary disease therapy [8]. Clinicians have reported that transradial access for cardiac catheterization significantly reduces the risk of local vascular complications and the incidence of postoperative adverse events [9, 10]. Additionally, patients preferred transradial to transfemoral access, as transradial access produces less discomfort and is associated with lower hospital costs [11, 12]. Considering that TACE via the femoral approach requires restrictive immobilization of the lower limb after the operation and results in delayed ambulation with a longer monitoring duration, transradial access technology is increasingly applied as an alternative to transfemoral access for angiography and intervention of other visceral organs and vessels, such as hepatic, uterine artery and peripheral interventions [13–15].

Several clinical studies have compared transfemoral and transradial access approaches for TACE [16–20], although reliable evidence for the extensive application of transradial access for TACE for the treatment of hepatic carcinoma is still lacking. Indeed, catheterization via a radial approach was reported to require higher technical skills with a decreased success rate [10], and patients could be exposed to a higher dose of radiation than with transfemoral access [21], which might make clinicians reluctant to use a radial approach for TACE. Thus, the application of transradial access remains controversial. The present research focused on the intraoperative technical outcomes and postoperative clinical parametric data of transradial access as an alternative to traditional transfemoral access for TACE in HCC patients and is intended to provide a reference for its clinical application.

## Methods

### Study population

Patients with primary HCC from the Second Affiliated Hospital of Chongqing Medical University were retrospectively assessed from 2018 to 2021. TACE treatments, including transradial or transfemoral access, were performed on the patients in this study. The TACE intervention approach was decided by the patients after they deliberated with the attending physician, and written informed consent was obtained from all patients prior to TACE treatment. The present study was designed and conducted in accordance with the principles of the Helsinki Declaration and was approved by the ethics committees of the involved hospital.

### Inclusion and exclusion criteria

The inclusion criteria were as follows: (1) age ≥ 18 years; (2) diagnosed primary HCC; (3) patients with unresectable HCC with no plan for further resection within one month [22, 23]; (4) liver function graded as Child‒Pugh class A or B; (5) normal preoperative blood pressure; (6) successful TACE; and (7) complete intraoperative and postoperative follow-up records.

The exclusion criteria were as follows: (1) age < 18 years; (2) severe liver dysfunction and inability to tolerate TACE operation; (3) allergy to lipiodol or chemotherapeutic agents; (4) severe arterial disease; (5) systemic infection or complications with other severe diseases; (6) failed operation or death; and (7) absence of required parametric data or drop-out cases; (8) transferred patients or patients with combined hepatic procedures.

### Procedures and treatments

The modified Allen’s test was performed prior to transradial access to determine the patients’ suitability for vascular access [24]. Transradial access was performed by puncturing the radial artery with a 21-G needle after topical anesthesia with 1% lidocaine, and ultrasound guidance was used when necessary. Following successful arterial puncture, a 4F Glidesheath Slender (Merit Medical^®^, USA) vascular introducer sheath was placed over a 0.021-inch microwire at the puncture site, thus allowing the use of a catheter. After insertion, a mix of 2.5 mg of verapamil, 2 mL of 2% lidocaine, and 2000 IU of heparin was injected via the introducer sheath to prevent vasospasm and avoid clot formation. A 4F × 125 cm Ultimate 1 Performa catheter was advanced into the abdominal aorta over a 0.038-inch × 180 cm Glidewire with a 1.5 mm J-tip. The inserted 4F catheter was then advanced to the celiac and/or superior mesenteric artery, depending on the target vessel to be embolized. Superselective catheterization was performed with a 2.7-F microcatheter advanced into the hepatic artery that was feeding the tumours. Embolization was performed with an emulsion consisting of pirarubicin, lobaplatin, and lipiodol injection. The doses of chemotherapeutic agents used for embolization depended on the conditions of the tumour and patients. In addition, microspheres (HENGRUI Medical^®^, China) were injected to enhance the embolization efficacy according to the sizes of the tumour vessels.

Transfemoral access was performed with a 4F introducer sheath (COOK Medical^®^, USA) advanced into the right common femoral artery. A 4F × 80 cm catheter was utilized to select the celiac and/or superior mesenteric artery for catheterization. In addition, superselective catheterization and chemoembolization were performed by placing a 2.7-F microcatheter into the target vessels feeding the tumours.

Haemostatic devices were used after TACE. For patients who underwent the RA route approach, a manual haemostasis device (Radiquick^®^, China) was utilized on the access site for 2 ~ 4 h without bed rest restriction. In the FA route group, the included subjects were administered an electronic haemostasis device (Efinger^®^, China) for 6 ~ 8 h with bedrest. Patients in both groups received routine preoperative and postoperative treatments in addition to RA- or FA-route TACE. Symptomatic treatments were routinely carried out for postoperative syndromes.

### Data collection

General patient information and basic parameters, including age, sex, HBV infection, liver function and tumour conditions, were collected prior to the TACE procedure. Intraoperative technical outcomes, such as operation duration, superselection, hepatic arterial spasm, and dose of chemotherapeutic agent, were quantitatively compared. Hepatic arterial spasm was recorded when significant visible segmental stenosis of the hepatic artery under DSA occurred during TACE. The efficacy and safety of the 2 approaches were compared based on the tumour response according to the modified Response Evaluation Criteria in Solid Tumours (mRECIST) for HCC [25] and adverse events. The evaluation of mRECIST was performed by 2 investigators independently. For the enrolled subjects with inconsistent mRECIST assessment, the results were decided through group discussion to reach a final consensus. Moreover, other parametric data, such as postoperative liver function, length of stay (LOS), visual analogue scale (VAS) score of abdominal pain, and cost of hospitalization (Chinese Yuan, CNY), were also quantitatively analysed. Liver function was indexed to the peak values within 7 postoperative days. The LOS was calculated based on inpatients receiving TACE treatment only and patients were discharged until the absence of postoperative adverse events. All included subjects would be stratified and compared.

### Statistical analysis

The analysis for continuous variables was performed with Student’s *t test*, and the results are presented as the means with standard error of the mean (SEM). Categorical variables were compared by employing χ^2^ or Fisher’s exact test. A *P* value < 0.05 was considered a statistically significant difference between the two groups. All data manipulation and statistical analysis were accomplished with IBM SPSS Statistics for Windows Version 22.0.

## Results

### Patient characteristics

**Fig. 1:**
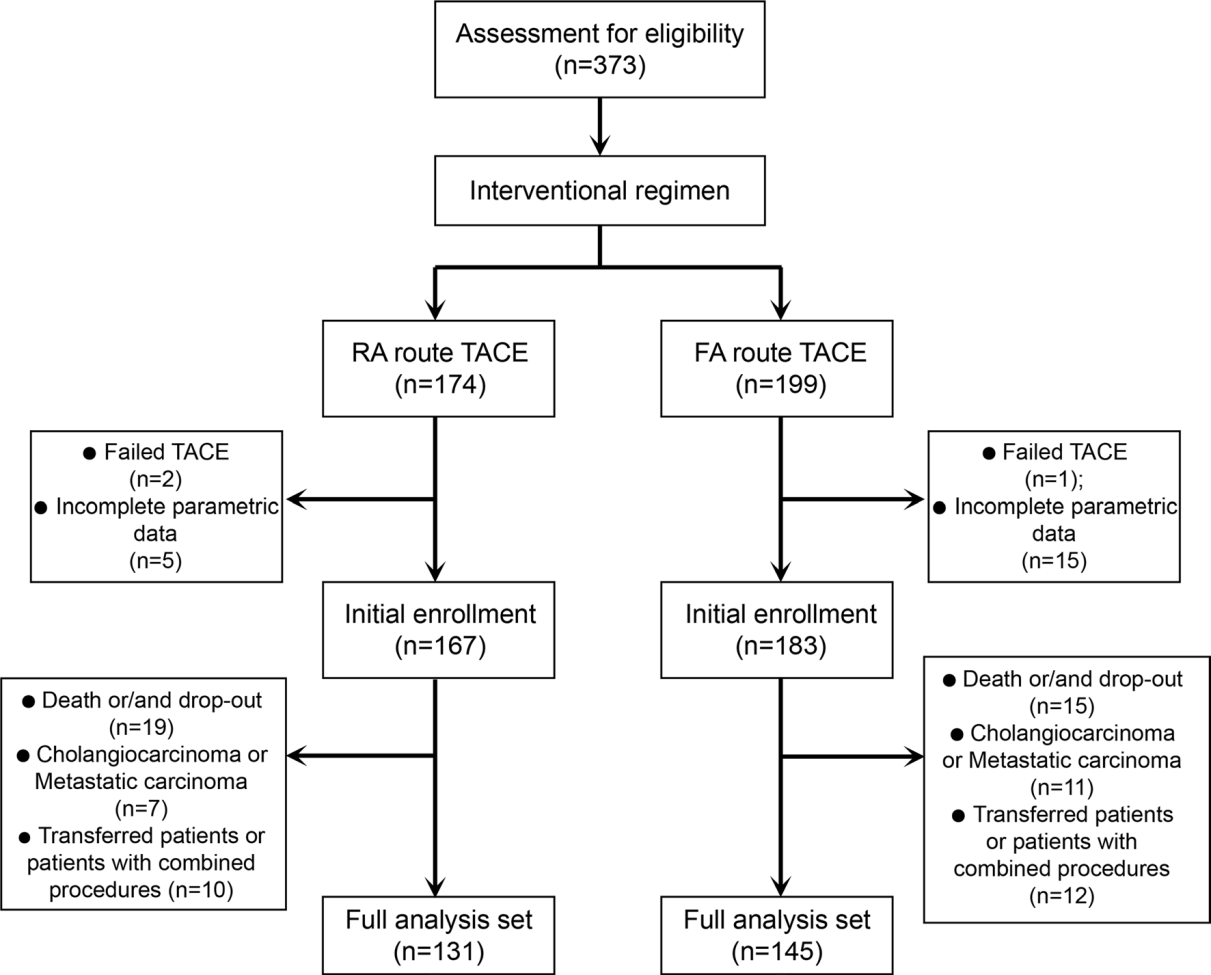
Patient flow chart. TACE, transarterial chemoembolization

In total, 373 patients were retrospectively assessed. Three patients were excluded for failed TACE, and 20 patients were excluded on account of incomplete parametric data. In addition, another 34 deaths or drop-out cases and 18 cholangiocarcinoma or metastatic carcinoma cases were excluded. We also excluded the 22 transferred patients and the subjects with combined hepatic procedures. Finally, 276 subjects were included in the present study, with 131 patients in the RA route group and 145 in the FA route group (Fig. 1). The demographic and baseline characteristics are summarized in Table 1. The baselines of the two groups were consistent, including the patients’ general information (age and sex), preoperative liver function indices, and tumour-associated parameters.

**Table 1:** Baseline characteristics of included patients

Item	RA route	FA route	*P*
Number of patients	131	145	
*Gender [n]*			
Male	117	132	0.687
Female	14	13	
Age			
*HBV [n]*			
Absent	14	17	0.849
Present	117	128	
*HCV [n]*			
Absent	129	140	0.451
Present	2	5	
*Alcohol abuse [n]*			
Absent	129	140	0.451
Present	2	5	
*Nodule number [n]*			
1	58	62	0.809
≥ 2	73	83	
*Maximum tumor size (cm)*			
≤ 5	74	72	0.278
> 5	57	73	
*Tumor locations*			
Left lobe	15	17	0.713
Right lobe	87	90	
Mixed or diffused	29	38	
*Child–Pugh [n]*			
A	114	130	0.573
B	17	15	
*AFP*			
≤ 400 ng/dL	94	88	0.057
> 400 ng/dL	37	57	
*Pre-operative liver function*			
TBIL (μmol/L)	15.26 ± 0.78	27.34 ± 11.63	0.325
ALT (U/L)	40.29 ± 2.23	48.14 ± 4.26	0.115
AST (U/L)	54.72 ± 5.19	73.41 ± 12.71	0.191

### Technical outcomes

**Fig. 2:**
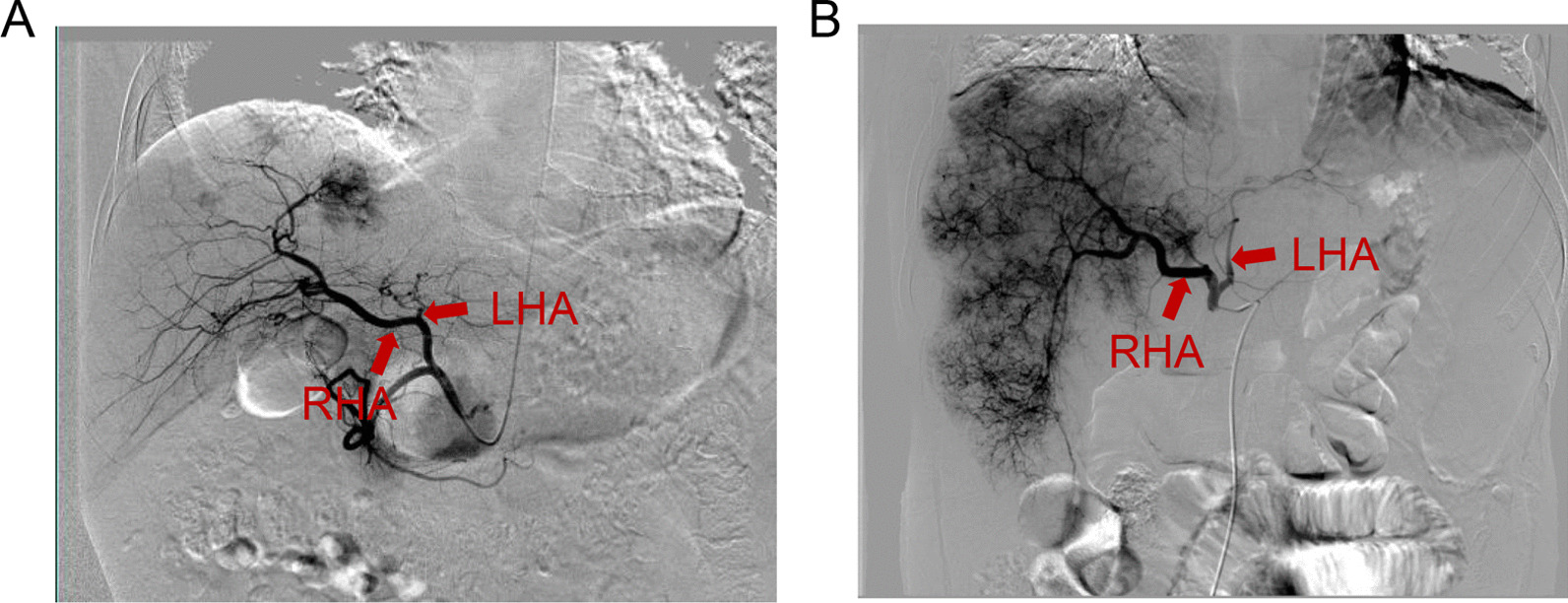
Representative pictures under DSA during TACE regarding the **A** RA route and **B** FA route. RHA, right hepatic artery; LHA, left hepatic artery

To compare the technical outcomes between the two routes, we investigated the intraoperative parametric data, including operation time, rate of superselection, hepatic arterial spasm and puncture site haematoma, and consumption of chemo-dose. All parameters were intraoperatively recorded, and representative images for the RA and FA route groups are presented in Fig. 2. The operation duration of the RA group was 84.86 ± 2.09 min, similar to that of the FA group (80.1 ± 2.37 min). Additionally, the doses of chemotherapeutic agents and rates of superselection and puncture site haematoma were also similar (Table 2). Interestingly, no hepatic arterial spasm was observed in the RA group, but 10 subjects were observed in the FA route group, indicating that the rate of hepatic arterial spasm was significantly higher in the FA route group (*P* = 0.002) (Table 2).

**Table 2:** Intraoperative technical outcomes of TACE patients

Item	RA-route	FA-route	*P*
Number of patients	131	145	
Operation time (min)	84.86 ± 2.09	80.61 ± 2.37	0.184
*Superselection*			
Yes	131	143	0.499
No	0	2	
*Rate of arterial spasm (%)*			
Yes	0	10	**0.002**
No	131	135	
*Rate of puncture site hematoma (%)*			
Yes	0	2	0.499
No	131	143	
*Consumption of chemo-dose (ml)*			
Lipiodol	9.83 ± 0.28	10.30 ± 0.37	0.336
Microsphere	2.60 ± 0.39	3.69 ± 0.46	0.075
Pirarubicin	36.87 ± 2.04	33.97 ± 1.55	0.253
Lobaplatin	18.02 ± 1.71	21.94 ± 1.71	0.107

The bold numbers of *P*-value represent statistical differences

### Efficacy

**Fig. 3:**
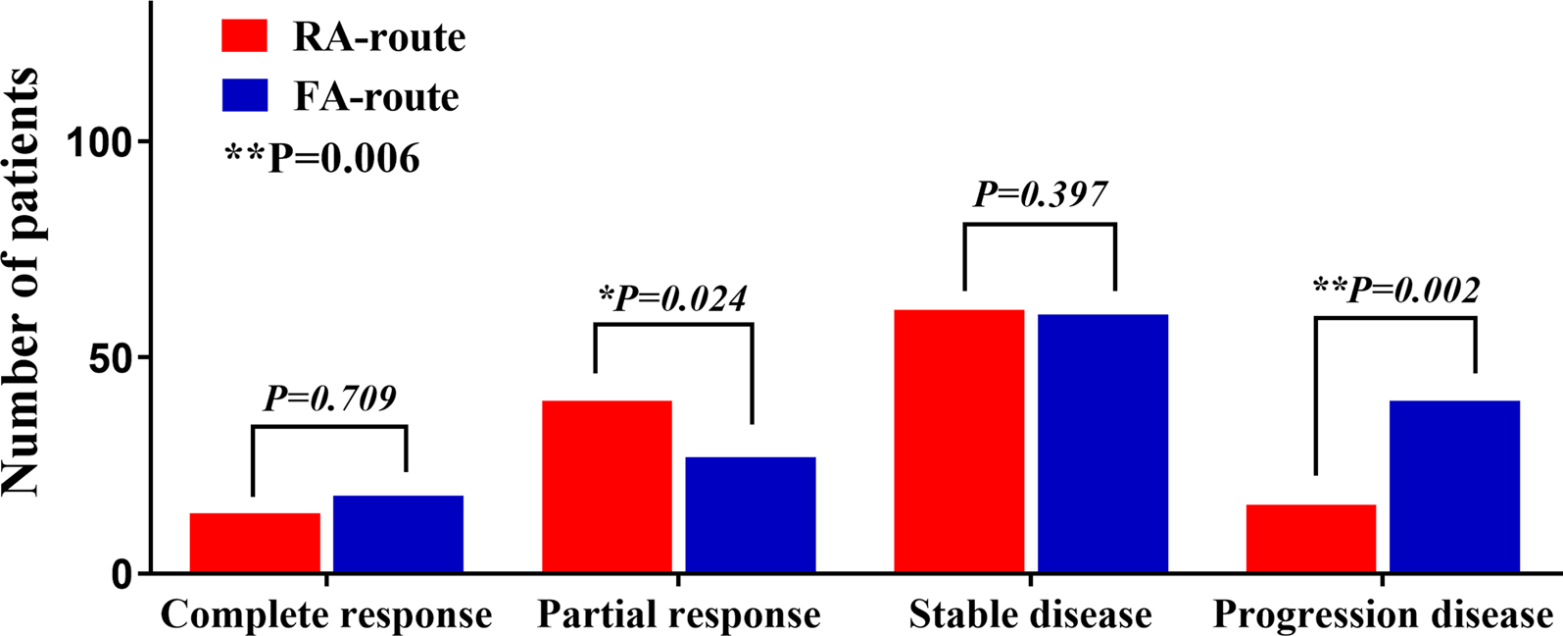
Assessment of the tumour response after TACE based on mRECIST guidelines for each group

**Fig. 4:**
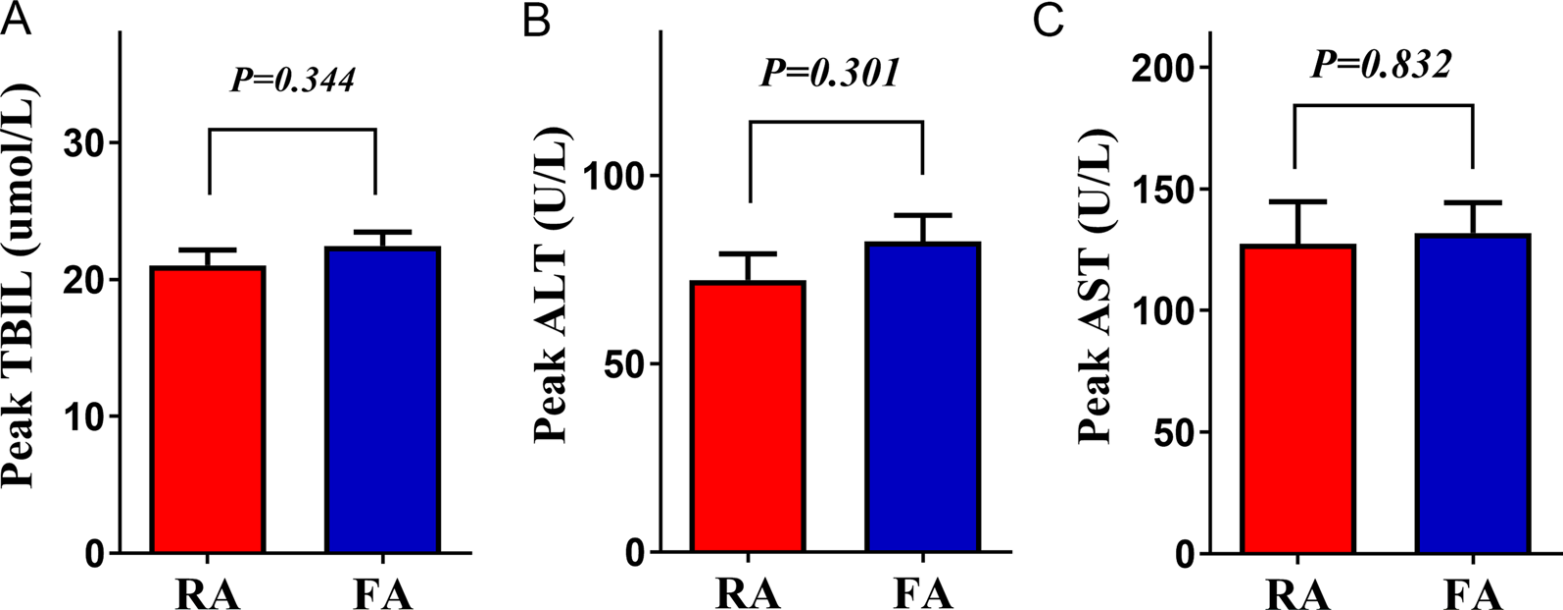
The peak values of liver function indices, including **A** TBIL, **B** ALT and **C** AST, within 7 days following the operation for patients in each group. TBIL, total bilirubin; ALT, alanine aminotransferase; AST, aspartate aminotransferase

**Fig. 5:**
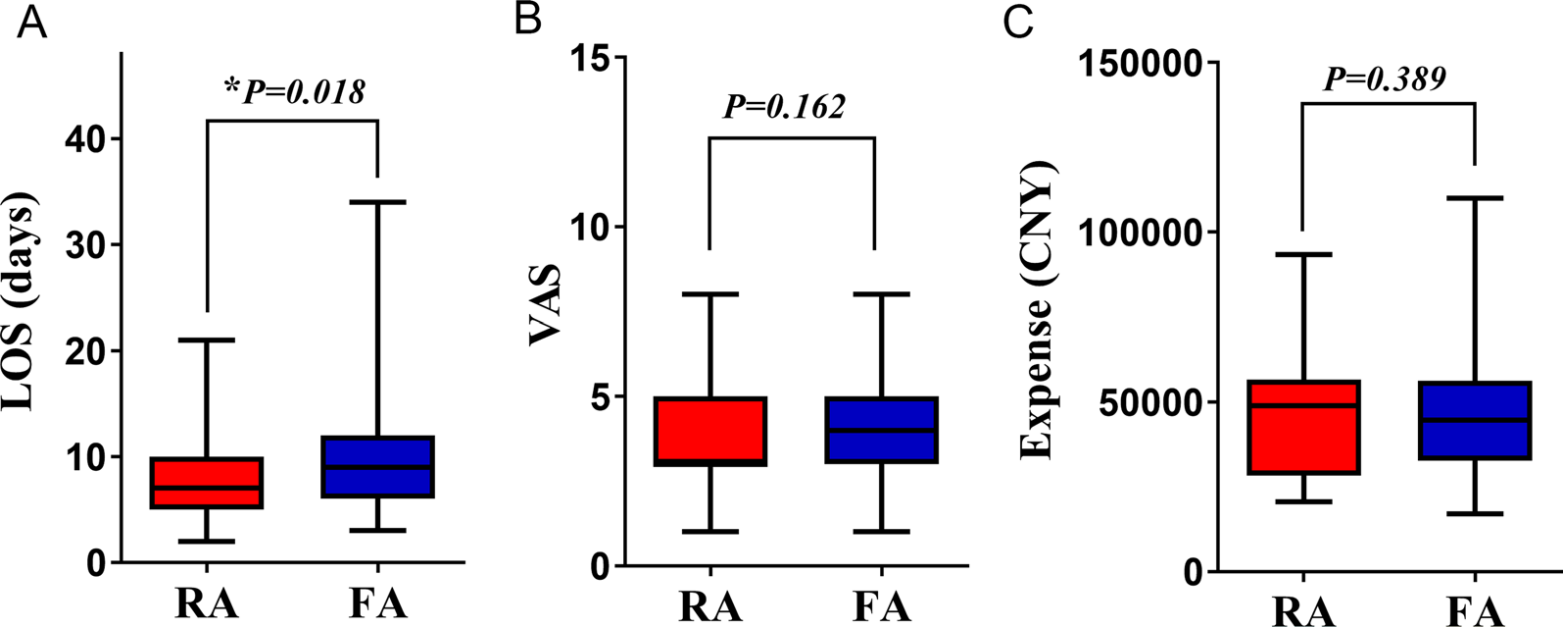
Hospitalization evaluation of the RA route and FA route groups compared by **A** LOS, **B** VAS pain scores, and **C** hospitalization expense for each group. LOS, length of stay; VAS, visual analogue scale

We evaluated the efficacy of TACE according to the mRECIST guidelines for primary HCC. The partial response rate in the RA route group was significantly higher than that in the FA route group (30.5% vs. 18.6%, *P* = 0.024). Conversely, more patients in the progression stage were observed in the FA route group after TACE than in the RA route group (12.2% vs. 27.6%, *P* = 0.002) (Fig. 3). These data suggest that RA-route TACE is more efficient than FA-route TACE. Postoperative liver functions indicated by the peak values of related indices TBIL (21.03 ± 1.13 vs. 22.45 ± 1.00 μmol/L, *P* = 0.344), ALT (72.21 ± 6.93 vs. 82.43 ± 6.99 U/L, *P* = 0.301), and AST (127.5 ± 17.25 vs. 131.9 ± 12.43 U/L, *P* = 0.832) were also compared between the two groups, and no significant intragroup differences were revealed (Fig. 4). However, an obviously shorter LOS (8.42 ± 0.36 vs. 9.63 ± 0.35 days, *P* = 0.018) was observed in patients who received RA-route TACE, while the VAS score (3.84 ± 0.14 vs. 4.10 ± 0.13, *P* = 0.162) and hospitalization expenses (45,072 ± 1506 vs. 46,890 ± 1472 CNY, *P* = 0.389) were similar to those of patients receiving FA route TACE (Fig. 5).

### Safety

The incidence rates of adverse events (AEs) commonly seen in the postoperative period are listed in Table 3. For general AEs, the occurrence of fever was significantly reduced in the RA route group (33.6% vs. 52.4%, *P* = 0.002). In addition, fewer patients suffered nausea (30.5% vs. 53.1%) and vomiting (13.7% vs. 24.8%) in the RA route group than in the FA route group. In summary, the total proportion of patients with adverse events in the RA group was 72.5% versus 84.1% in the FA group, with a *p* value of 0.027. Thus, transradial access is a safe and practical choice for hepatic intervention.

**Table 3:** Postoperative adverse events of included TACE patients

Item	RA-route (131)	FA-route (145)	*P*
*General events [n (%)]*			
Fever	44 (33.6)	76 (52.4)	**0.002**
Fatigue	17 (13.0)	29 (20.0)	0.145
*Gastrointestinal events [n (%)]*			
Nausea	40 (30.5)	77 (53.1)	** < 0.001**
Vomiting	18 (13.7)	36 (24.8)	**0.022**
Constipation	9 (6.9)	9 (6.2)	0.999
Obvious abdominal pain	61 (46.6)	79 (54.5)	0.147
Ascites	6 (4.6)	4 (3.0)	0.525
Diarrhea	2 (1.5)	0 (0)	0.224
*Others [n (%)]*			
Wound infection	0 (0)	0 (0)	–
Intrahepatic infections	0 (0)	3 (2.1)	0.249
Hypertension	10 (7.6)	7 (4.8)	0.453
Urine retention	1 (0.8)	2 (1.4)	0.999
Coagulation	0 (0)	2 (1.4)	0.499
Vasovagal reaction	0 (0)	0 (0)	–
Pancreatitis	0 (0)	0 (0)	–
Total cases with AE [n (%)]	95 (72.5)	122 (84.1)	**0.027**

The bold numbers of *P*-value represent statistical differences

### Stratified analysis

To further investigate the potential impacts of the differences mentioned above, including hepatic arterial spasm, the rate of partial response and progression, and adverse events, we conducted stratified analysis regarding tumour size and AFP. The results indicated that the RA route presented a lower rate of arterial spasm (*P* = 0.013) and tumour progression (*P* = 0.007) in tumours < 5 cm and a higher rate of partial response (*P* = 0.019) in tumours > 5 cm. Meanwhile, the RA route revealed superiorities in patients with lower AFP and subjects in the TACE only stratified group but not in subjects with high AFP (Table 4). On the other hand, according to the results of stratified intergroups, patients with tumours < 5 cm and lower AFP more or less presented superiorities in the LOS, tumour response and AEs (Table 4).

**Table 4:** Stratified analysis based on the results with differences

Stratified items	Rate of hepatic arterial spasm			LOS			mRECIST (PR)			mRECIST (progression)			Total subjects with AEs		
	RA route	FA route	*P*	RA route	FA route	*P*	RA route	FA route	*P*	RA route	FA route	*P*	RA route	FA route	*P*
*Tumor size (cm)*															
≤ 5	0	6	**0.013**	7.64 ± 0.47	8.61 ± 0.38	0.111	17	12	0.408	4	15	**0.007**	48	55	0.148
> 5	0	4	0.131	9.42 ± 0.54	10.64 ± 0.57	0.134	23	15	**0.019**	12	25	0.119	47	67	0.177
*P*	0.999	0.532		**0.015**	**0.004**		**0.037**	0.670		**0.013**	0.094		**0.030**	**0.013**	
*AFP*															
≤ 400 ng/dL	0	7	**0.005**	8.26 ± 0.46	9.17 ± 0.47	0.168	31	16	**0.028**	8	19	**0.021**	62	72	**0.019**
> 400 ng/dL	0	3	0.276	8.81 ± 0.52	10.35 ± 0.53	0.051	9	11	0.611	8	21	0.170	33	50	0.999
*P*	0.999	0.740		0.503	0.108		0.402	0.999		0.071	0.057		**0.009**	0.485	

The bold numbers of *P*-value represent statistical differences

## Discussion

The intraoperative technical outcomes implied a lower rate of hepatic arterial spasm in the RA route group. Kiemeneij et al. [26] reported that radial artery spasm manifested when patients felt forearm pain or when there was resistance to the advancement of the intra-arterial equipment. In other words, arterial spasm may be attributed to the stimulation of catheterization and friction of the sheath against the vessel, leading to patient discomfort and obstruction of the embolization procedure. Similarly, for the hepatic artery, considering vascular anatomy, long-range catheterization and sheath movement, TACE could provoke visible hepatic artery spasm, as previously reported [27]. On the other hand, transfemoral access is the conventional approach for angiography and is a relatively easy operating procedure. Comparatively, transradial access technology is more challenging and calls for longer surgery duration, a greater level of proficiency [10, 21] and inevitably demands more advanced catheterization devices for its extensive application. The sheath, which is utilized for the transradial approach, is designed to be more applicable to the anatomy of liver vessels in terms of shape and length and requires gentler and more discreet operation when catheterizing compared with the catheters used for the transfemoral approach. These factors may reduce the incidence of hepatic arterial spasm in the RA route. The reduced proclivity to hepatic arterial spasm with the RA approach might contribute to tumour angiography and improve the subsequent chemoembolization of the vessels feeding the tumours, thus yielding superior efficacy after treatment. According to our results, the grades assessed in accordance with mRECIST determined that transradial access yielded preferable effectiveness to transfemoral access. 

Transradial access was confirmed to be a reliable alternative to transfemoral access chemoembolization and to be relatively safe. We revealed that the overall incidence of adverse events was significantly lower in the RA group than in the FA group. Fever, nausea and vomiting, the typical symptoms of postembolization syndrome [28], were less common in patients undergoing transradial access chemoembolization. This result may also be related to the abovementioned gentle procedure associated with transradial access and the design of the vascular devices used. A large randomized comparative study also reported that transradial access for coronary intervention achieved a lower incidence rate of entry site complications [29]. In our research, puncture site haematoma occurred in 2 patients in the FA route group, but no significant intragroup differences were found. Obvious abdominal pain was also equally frequent in the RA and FA groups, consistent with the approximated VAS scores. Other common complications, such as intrahepatic infection, hypertension and coagulation, were not different between the two groups.

Previous research describing the feasibility and efficacy of transradial access in TACE indicated that the procedure time was similar to that of transfemoral access [18–20], in accordance with our results. However, a systematic review that statistically pooled the results suggested that the overall procedure time was significantly longer in the transradial access group [30]. On the other hand, previous studies reported a risk of procedure failure despite no difference in results between the two access approaches [17, 18]. The present study omitted cases of incomplete or failed procedures to consider the efficacy and safety of the different access methods, eliminating the influence of procedural proficiency and heterogeneous circumstances. In addition, the high success rate of TACE via a radial approach is expected along with the technical progress of materials and increased procedural experience of clinicians. Moreover, the stratified analysis determined the superiority of the RA approach in different subgroups, especially for patients with tumours < 5 cm or lower AFP, indicating that the RA route may provide more potential benefits at certain stratifications. The results of stratified analysis also demonstrated that, to some extent, the subjects with tumours < 5 cm or lower AFP revealed better clinical outcomes after TACE, consistent with previous reports [31, 32]. Notably, the conclusions based on stratified analysis still need to be further verified, mainly because there are many relevant influencing factors, and the sample size of some subgroups was significantly smaller after stratification.

Patients who underwent TACE via a femoral arterial approach were required to remain immobilized in the supine position for at least 6 h after the operation to prevent bleeding and haematoma. Pressure haemostatic devices were applied in both TACE groups, but no bedrest was needed for subjects with the RA route, which caused delayed ambulation in the FA route group. Delayed ambulation necessitated a longer monitoring time and, thus, increased patient discomfort [16]. The results of a patient questionnaire also indicated that patients preferred transradial access to transfemoral access [19]. Both delayed ambulation and discomfort may contribute to gastrointestinal peristalsis disorder, which may also potentially increase the incidence of gastrointestinal AEs. On the other hand, for the LOS of clinical outcomes, patients in the RA route group had a significantly shorter LOS than those in the FA route group. The gentler procedure, earlier ambulation and fewer AEs may result in a shorter LOS, which was also confirmed in a previous study [16]. Nevertheless, TACE patients were generally discharged until the absence of AEs in our institution. Therefore, the overall LOS in our study seemed to be longer than usual. However, the main conclusion was that the shorter LOS in the RA route group did not fluctuate. Moreover, the hospitalization expenses were similar in both groups in the present study, in contrast with results reported in other studies [16, 17]. Costs likely differ across different institutions, causing discrepancies in hospitalization expense data. In addition, patients receiving TACE treatment are mainly charged in terms of operation-related costs, and the consumable materials and medicines are approximately accordant in both operation approaches, although the AE treatments were significantly more common in the FA route group. Perioperative management for patients was performed routinely according to the protocols at a predictable cost. Thus, TACE with transradial access is a practical alternative to traditional transfemoral access and does not increase the hospitalization cost.

This retrospective clinical study compared transradial and transfemoral access technology in hepatic carcinoma following the technical and clinical outcomes. However, the study has some limitations. Previous studies reported the fluoroscopy time during the procedure, which is an underlying factor associated with radiation dose and contrast volume [19, 33], and this parameter was absent in our evaluation. While we assessed the VAS score and postoperative complications, we did not assess patient preference for the access approach used for TACE treatment [34]. We hypothesized that hepatic arterial spasm might influence the tumour response after TACE, but due to the low incidence of hepatic arterial spasm, this speculation could not be addressed in the current study. However, this may be an interesting direction in future clinical research. Therefore, a large-scale randomized controlled trial with rigorous design is warranted to determine the feasibility and reliability of transradial access to replace transfemoral access for future hepatic interventions.

In conclusion, transradial access is a feasible alternative to transfemoral access which has less hepatic arterial spam with the help of advanced catheterization and has shorter hospitalization stays while reducing postoperative complications.

## Data Availability

The datasets used and/or analysed during the current study available from the corresponding author on reasonable request.

## References

[1] Forner A, Reig M, Bruix J (2018). Hepatocellular carcinoma. Lancet.

[2] EASL-EORTC clinical practice guidelines (2012). Management of hepatocellular carcinoma. J Hepatol.

[3] Heimbach JK, Kulik LM, Finn RS, Sirlin CB, Abecassis MM, Roberts LR (2018). AASLD guidelines for the treatment of hepatocellular carcinoma. Hepatology.

[4] Omata M, Cheng AL, Kokudo N, Kudo M, Lee JM, Jia J (2017). Asia-Pacific clinical practice guidelines on the management of hepatocellular carcinoma: a 2017 update. Hepatol Int.

[5] Seldinger SI (1953). Catheter replacement of the needle in percutaneous arteriography; a new technique. Acta Radiol.

[6] Varela-Cancelo A, Salgado-Fernández J, Calviño-Santos R, Bouzas-Zubeldía B (2020). Successful transfemoral management of transcatheter aortic valve embolization into left ventricle. Eur Heart J.

[7] Ogasawara S, Chiba T, Ooka Y, Kanogawa N, Motoyama T, Suzuki E (2018). A randomized placebo-controlled trial of prophylactic dexamethasone for transcatheter arterial chemoembolization. Hepatology.

[8] Sgueglia GA, Di Giorgio A, Gaspardone A, Babunashvili A (2018). Anatomic basis and physiological rationale of distal radial artery access for percutaneous coronary and endovascular procedures. JACC Cardiovasc Interv.

[9] Valgimigli M, Gagnor A, Calabró P, Frigoli E, Leonardi S, Zaro T (2015). Radial versus femoral access in patients with acute coronary syndromes undergoing invasive management: a randomised multicentre trial. Lancet.

[10] Agostoni P, Biondi-Zoccai GG, de Benedictis ML, Rigattieri S, Turri M, Anselmi M (2004). Radial versus femoral approach for percutaneous coronary diagnostic and interventional procedures; systematic overview and meta-analysis of randomized trials. J Am Coll Cardiol.

[11] Cooper CJ, El-Shiekh RA, Cohen DJ, Blaesing L, Burket MW, Basu A (1999). Effect of transradial access on quality of life and cost of cardiac catheterization: a randomized comparison. Am Heart J.

[12] Sciahbasi A, Fischetti D, Picciolo A, Patrizi R, Sperduti I, Colonna G (2009). Transradial access compared with femoral puncture closure devices in percutaneous coronary procedures. Int J Cardiol.

[13] Posham R, Biederman DM, Patel RS, Kim E, Tabori NE, Nowakowski FS (2016). Transradial approach for noncoronary Interventions: a single-center review of safety and feasibility in the first 1,500 cases. J Vasc Interv Radiol.

[14] Resnick NJ, Kim E, Patel RS, Lookstein RA, Nowakowski FS, Fischman AM (2014). Uterine artery embolization using a transradial approach: initial experience and technique. J Vasc Interv Radiol.

[15] Coscas R, de Blic R, Capdevila C, Javerliat I, Goëau-Brissonniere O, Coggia M (2015). Percutaneous radial access for peripheral transluminal angioplasty. J Vasc Surg.

[16] Wu T, Sun R, Huang Y, Wang Z, Yin X, Zhu Z (2015). Transradial arterial chemoembolization reduces complications and costs in patients with hepatocellular carcinoma. Indian J Cancer.

[17] Kis B, Mills M, Hoffe SE (2016). Hepatic radioembolization from transradial access: initial experience and comparison to transfemoral access. Diagn Interv Radiol.

[18] Shiozawa S, Tsuchiya A, Endo S, Kato H, Katsube T, Kumazawa K (2003). Transradial approach for transcatheter arterial chemoembolization in patients with hepatocellular carcinoma: comparison with conventional transfemoral approach. J Clin Gastroenterol.

[19] Iezzi R, Pompili M, Posa A, Annicchiarico E, Garcovich M, Merlino B (2017). Transradial versus transfemoral access for hepatic chemoembolization: intrapatient prospective single-center study. J Vasc Interv Radiol.

[20] Yamada R, Bracewell S, Bassaco B, Camacho J, Anderson MB, Conrad A (2018). Transradial versus transfemoral arterial access in liver cancer embolization: randomized trial to assess patient satisfaction. J Vasc Interv Radiol.

[21] Mercuri M, Mehta S, Xie C, Valettas N, Velianou JL, Natarajan MK (2011). Radial artery access as a predictor of increased radiation exposure during a diagnostic cardiac catheterization procedure. JACC Cardiovasc Interv.

[22] Yoon JH, Park JW, Lee JM (2016). Noninvasive diagnosis of hepatocellular carcinoma: elaboration on Korean liver cancer study group-national cancer center Korea practice guidelines compared with other guidelines and remaining issues. Korean J Radiol.

[23] Chinese College of Interventionalists, Chinese Medical Doctor Association. [Chinese Clinical Practice Guidelines for transarterial chemoembolization of hepatocellular carcinoma]. Zhonghua Gan Zang Bing Za Zhi. 2019;27(3):172–81. 10.3760/cma.j.issn.1007-3418.2019.03.003 PMC12814237 30929333

[24] Pinter L, Cagiannos C, Ruzsa Z, Bakoyiannis C, Kolvenbach R (2007). Report on initial experience with transradial access for carotid artery stenting. J Vasc Surg.

[25] Lencioni R, Llovet JM (2010). Modified RECIST (mRECIST) assessment for hepatocellular carcinoma. Semin Liver Dis.

[26] Kiemeneij F, Vajifdar BU, Eccleshall SC, Laarman G, Slagboom T, van der Wieken R (2001). Measurement of radial artery spasm using an automatic pullback device. Catheter Cardiovasc Interv.

[27] Xia J, Ren Z, Ye S, Sharma D, Lin Z, Gan Y (2006). Study of severe and rare complications of transarterial chemoembolization (TACE) for liver cancer. Eur J Radiol.

[28] Khalaf MH, Sundaram V, AbdelRazek MM, Shah R, Khosla A, Jackson K (2019). A predictive model for postembolization syndrome after transarterial hepatic chemoembolization of hepatocellular carcinoma. Radiology.

[29] Kiemeneij F, Laarman GJ, Odekerken D, Slagboom T, van der Wieken R (1997). A randomized comparison of percutaneous transluminal coronary angioplasty by the radial, brachial and femoral approaches: the access study. J Am Coll Cardiol.

[30] Chen YY, Liu P, Wu YS, Lin H, Chen X (2018). Transradial vs transfemoral access in patients with hepatic malignancy and undergoing hepatic interventions: a systematic review and meta-analysis. Medicine.

[31] Bannangkoon K, Hongsakul K, Tubtawee T, McNeil E, Sriplung H, Chongsuvivatwong V (2018). Rate and predictive factors for sustained complete response after selective transarterial chemoembolization (TACE) in patients with hepatocellular carcinoma. Asian Pac J Cancer Prev.

[32] Prateepchaiboon T, Chang A, Pungpipattrakul N, Akarapatima K, Rattanasupar A, Songjamrat A (2022). Factors affecting prognosis in hepatocellular carcinoma patients post-transarterial chemoembolization. Indian J Gastroenterol.

[33] Sciahbasi A, Frigoli E, Sarandrea A, Rothenbühler M, Calabrò P, Lupi A (2017). Radiation exposure and vascular access in acute coronary syndromes: the RAD-matrix trial. J Am Coll Cardiol.

[34] Liu LB, Cedillo MA, Bishay V, Ranade M, Patel RS, Kim E (2019). Patient experience and preference in transradial versus transfemoral access during transarterial radioembolization: a randomized single-center trial. J Vasc Interv Radiol.

